# Non-Specific Abdominal Pain and Air Pollution: A Novel Association

**DOI:** 10.1371/journal.pone.0047669

**Published:** 2012-10-31

**Authors:** Gilaad G. Kaplan, Mieczyslaw Szyszkowicz, Jakub Fichna, Brian H. Rowe, Eugeniusz Porada, Renaud Vincent, Karen Madsen, Subrata Ghosh, Martin Storr

**Affiliations:** 1 Division of Gastroenterology, University of Calgary, Calgary, Alberta, Canada; 2 Departments of Medicine, University of Calgary, Calgary, Alberta, Canada; 3 Community Health Sciences, University of Calgary, Calgary, Alberta, Canada; 4 Environmental Health Sciences and Research Bureau, Health Canada, Ottawa, Ontario, Canada; 5 Department of Emergency Medicine and School of Public Health, University of Alberta, Edmonton, Alberta, Canada; 6 Department of Medicine, Division of Gastroenterology, University of Alberta, Edmonton, Alberta, Canada; 7 Department of Biomolecular Chemistry, Medical University of Lodz, Lodz, Poland; 8 Department of Medicine, Ludwigs-Maximilians University, Munich, Germany; University of Montreal, Canada

## Abstract

**Background:**

We studied whether short-term exposure to air pollution was associated with non-specific abdominal pain in epidemiologic and animal studies.

**Methods:**

Patients visiting the emergency department with non-specific abdominal pain were identified in Edmonton (1992 to 2002, n = 95,173) and Montreal (1997 to 2002, n = 25,852). We calculated the daily concentrations for ozone (O_3_), nitrogen dioxide (NO_2_), sulfur dioxide (SO_2_), carbon monoxide (CO), and particles <10 (PM_10_) or <2.5 (PM_2.5_) µm. A case crossover study design was used to estimate the odds ratio (OR) and 95% confidence interval (CI) associated with an increase in the interquartile range of the air pollutants. We investigated differential effects by age and sex. Mice were gavaged with urban particle extracts. In animal models, colonic motility was tested, and visceral abdominal pain was measured using a writhing test, and behavioral response to oil of mustard and neostigmine. Motility and pain was measured acutely (1.5 hours after gavage) and chronically (7-days and 21-days after gavage).

**Results:**

Emergency department visits for non-specific abdominal pain were primarily by women between the ages of 15–24 years. Individuals aged 15 to 24 years were at increased risk of non-specific abdominal pain in Edmonton (same day CO: OR = 1.04, 95% CI = 1.02–1.06; and NO_2_: OR = 1.06, 95% CI = 1.03–1.09). The risk of air pollution among 15–24 year olds in Montreal was significantly positive (same day CO: OR = 1.11, 95% CI = 1.05–1.17; NO_2_: OR = 1.09, 95% CI = 1.01–1.16; SO_2_: OR = 1.17, 95% CI = 1.10–1.25; PM_2.5_: OR = 1.09, 95% CI = 1.04–1.15). Abdominal pain was increased by an acute gavage of pollution extract but not to chronic exposure to pollutants. Colonic transit was delayed following chronic but not acute exposure with the pollutants.

**Conclusions:**

Epidemiological and animal data suggest that short-term exposure to air pollution may trigger non-specific abdominal pain in young individuals.

## Introduction

Abdominal pain is the second most common diagnosis (4.2%) claimed by emergency department physicians in the USA. In many cases patients are diagnosed with non-specific abdominal pain, which refers to abdominal pain that is not explained during acute assessment. [1], [2] While many patients diagnosed with non-specific abdominal pain are asymptomatic at a later outpatient follow-up [3], [4], [5], a subset of these patients require recurrent admissions. [6], [7], [8] Consequently, non-specific abdominal pain is an important clinical presentation associated with impairment in quality of life for patients and significant costs to the health care system. [7], [8]

The underlying cause of non-specific abdominal pain is likely multifactorial. non-specific abdominal pain may be due to gastrointestinal diseases such as Crohn's [9] and celiac [10] diseases that are diagnosed after the patient is discharged from the emergency department. Additionally, some patients experience a transient illness such as gastrointestinal infection. Cases of non-specific abdominal pain may also represent a functional disorder such as irritable bowel syndrome. [11] Despite extensive research into the causes of non-specific abdominal pain, risk factors are not known and thus, exploration of triggers of non-specific abdominal pain is warranted.

Air pollution directly effects pulmonary diseases (e.g. asthma) and has been associated with non-pulmonary diseases including cancer and strokes. [12], [13], [14] Recently, studies have indicated that air pollution may affect gastrointestinal disorders such as the inflammatory bowel diseases and appendicitis. [15], [16] Air pollutants can cause systemic effects such as cardiovascular [17] or liver metabolic [18] changes after primary deposition and direct effects in the respiratory compartment. [19] However, most inhaled particles deposited in the nasopharyngeal compartment and airways are removed through mucociliary clearance and swallowed within a day [20] and thus, gastrointestinal effects may be due to direct effects of particles. [19]

Consequently, we investigated whether acute exposure to air pollutants was associated with emergency department visits for non-specific abdominal pain by: 1) evaluating the effect of short-term ambient air pollution exposure in a human population; 2) replicating this epidemiological study in a second population; and 3) exploring the biological plausibility in *in vitro and in vivo* models.

## Methods

### Phase 1: Epidemiological Studies

#### Study Population

As described previously [21], the administrative database study was approved by the individual research ethics boards at each participating institution and the data were transferred to the Health Canada following de-identification. No patient contact was made and patients could not be traced. The International Classification for Disease 9^th^ revision was used to identify patients who were discharged from an emergency department with a primary diagnosis of non-specific abdominal pain (789.0×). [2] First, we identified a population-based sample of 95,173 individuals who visited one of 5 emergency department in Edmonton for non-specific abdominal pain from 1992–2002. Second, we identified a replication study population of 25,852 individuals who visited a downtown emergency department in Montreal for non-specific abdominal pain from 1997–2002. Three digit postal codes were used to restrict the study population to residents of the city of Edmonton or Montreal. While the study population from Edmonton was population-based and included all ages, pediatric emergency department visits were underrepresented in Montreal because of the immediate proximity of a Pediatric Hospital. Thus, for the study population from Montreal the analysis was restricted to individuals over the age of 15 years.

#### Air Pollution and Meteorological Data

The air pollutants measured included sulfur dioxide (SO_2_), nitrogen dioxide (NO_2_), carbon monoxide CO, ozone (O_3_), and particulate matter characterized by an aerodynamic diameter of ≤10 µm (PM_10_) and of ≤2.5 µm (PM_2.5_). The data on air pollution were supplied by Environment Canada from fixed monitoring stations in Edmonton and Montreal. Environment Canada also supplied hourly data for temperature and relative humidity. For each air pollutant and weather variable, we obtained data on 24 measurements carried out at the monitoring station at hourly intervals. If more than 6 measurements were missing for a specific day, than the data for this monitor was considered as missing. An average was calculated for each monitor to represent daily mean value and average among monitors was used to represent ambient exposure across the city. [22] The distribution of air pollution and weather monitors for both cities can be found in Environment Canada's website. [23] A detailed description of air pollutant and weather measurements, monitor distribution, and correlations among monitors has been previously reported. [24]

#### Statistical Analysis

We applied a case crossover study design, which is an adaptation of the case–control study whereby cases serve as their own controls. [25] A time-stratified design was used to select referent periods, whereby the referent periods were selected from the same day of the week, month and year as the case's date of hospital visit. Because within-individual comparisons were made, confounding due to time-independent risk factors (e.g. genetics) was reduced. Similarly, the matching of control to case periods by day of week controlled for the influence of “day of week” effects. [25]

We used a conditional logistic regression (subroutine PHREG in SAS) to model the air pollutants and 6 components for natural splines for temperature and relative humidity. Estimates were expressed as the odds ratio (OR) with 95% confidence intervals (CI) associated with an increase in the interquartile range of the air pollutants (SO_2_, NO_2_, CO, O_3_, PM_10_, and PM_2.5_) after adjusting for temperature and relative humidity. Control for potentially non-linear effects of weather was achieved by including natural spline functions of temperature and relative humidity. The interquartile range was calculated based on the daily mean concentration of each air pollutant during the study period (Table S1). Analyses were performed separately for Edmonton and Montreal. Additionally, the analysis was stratified by: age, categorized as <15 (only Edmonton), 15–24, 25–34, 35–44, 45–64, and >64 years. Different time lags for pollution and weather exposures were used in our analyses including same day, one day before admission (i.e. 1-day lag), and two days before admission (i.e. 2-day lag). Among individuals 15 to 24 years of age we examined for effect modification by sex by incorporating an interaction term (e.g. PM_2.5_ X sex) into the model. All statistical analyses were conducted in SAS (version 8, SAS, Cary, North Carolina, US).

### Phase 2: *In Vitro & In Vivo* Studies

#### Animals

Male Swiss albino mice (CD1, Charles River, Canada), weighing 24–26 g, were used. The animals were housed at a constant temperature (22°C) and maintained under a 12-h light/dark cycle with free access to laboratory chow and tap water. Animal use was approved by the University of Calgary Animal Care Committee.

#### Particulate Matter Preparation

The urban particles EHC-6802 consisted of a blend of total suspended particulate matter recovered from filters of the single-pass air purification system at the Environmental Health Centre (Tunney's Pasture, Ottawa, ON) in 1996, 1998, 2000, and 2002, as described previously. [26] Particles were mechanically sieved using a 36 µm mesh filter, and combined in equal proportions. EHC-6802 was recovered at the same site and manner as the urban particles EHC-93, and chemical characterization of the EHC-6802 material has confirmed that it was equivalent to the EHC-93 material. [26]

#### Experimental Design

Animals were randomly assigned to two groups with the same number of animals per treatment group: control (oral tap water, 200 µl/animal, once daily; n = 8) or EHC-6802 (oral 360 µg/200 µl tap water/animal, once daily; n = 8). Gavages were given once daily and experiments were performed in the following protocols: 1) 1.5 h after the gavage; 2) daily for 7 days; and 3) daily for 21 days.

#### Isolated smooth muscle strips

Full-thickness segments (1 cm) of ileum and distal colon were resected. The preparations were mounted between two platinum electrodes and placed longitudinally in separate organ baths as described previously. [27] One end of each preparation was attached to the bottom of the organ bath, while the other end was connected to a FT03 force displacement transducer (Grass Technologies, West Warwick, RI, USA). Changes in tension were amplified by a P11T amplifier (Grass Technologies) and recorded on a computer using PolyView software (Polybytes, Cedar Rapids, Iowa).

Electrical field stimulation (EFS; 8 Hz; 60 V; pulse duration 0.5 ms; train duration 10 sec) was applied by a S88X stimulator (Grass Technologies). EFS of isolated smooth muscle strips caused twitch contractions, which were of cholinergic neuronal origin as they were virtually abolished by atropine (10^−6^ M) or TTX (10^−6^ M) (data not shown). In separate experiments the response of ileal and colonic muscle strips to direct muscular activation with stepwise addition of bethanechol (10^−7^ M–3×10^−5^ M) was recorded.

EHC-6802 urban ambient particles were added cumulatively into the organ baths and effects on the EFS-induced contractions were recorded. The mean amplitude of 4 successive twitch contractions was used as an internal control. Changes in contractions were reported as the percentage of the internal control. In control experiments the effects of the vehicle (tap water) were tested to control against possible effects on contractility by adding vehicle..

In separate experiments the response of ileal and colonic muscle strips to (i) neuronal electrical stimulation and (ii) direct muscular activation with stepwise addition of bethanechol (10^−7^ M–3×10^−5^ M) was recorded following 90 min, 7 days and 21 days following oral treatment with EHC-6802 particles.

#### Colonic expulsion test

Distal colonic expulsion was measured as reported previously. [27] 1.5 hours after receiving tap water (control) or EHC-6802 particle suspension (n = 5–10), a glass bead (2 mm) was inserted 2 cm into the distal colon and the time to bead expulsion was determined.

#### Behavioural Pain Responses

Behavioural pain responses were tested in 3 models used to study abdominal visceral pain responses (n = 5–10). The writhing test was performed as described by Gach et al. [28] Ninety minutes after oral administration of tap water (control group) or EHC-6802 particle suspension, mice received an intraperitoneal (i.p.) injection (10 ml/kg) of acetic acid solution (0.5%). The total number of writhes was counted 5 minutes after acetic acid injection, during three periods lasting 5 minutes each. The writhing response, considered as a nociceptive behaviour, was characterized by elongation of the body and the development of tension in the abdominal muscles and hind paws.

Behavioural responses to intracolonic (i.c.) oil of mustard (OM) and i.p. neostigmine were determined based on Laird et al. [29] and Eijkelkamp et al. (n = 5–10). [30] For the assessment of the OM-induced pain behaviours, 50 µl of OM (1% in 70% ethanol-30% saline) was administered i.c. under isoflurane anaesthesia. Petroleum jelly was applied to the perianal area to avoid stimulation of somatic areas. After 5 min of recovery, spontaneous behaviours were videotaped for analysis by an observer blinded to the experimental conditions.

In a separate experiment, neostigmine (2.5 µg/kg) or vehicle was administered i.p. and the pain-related behaviours were counted over a 10 minute period after the injection. Postures defined as pain-related behaviours: 1) licking of the abdomen, 2) stretching the abdomen, 3) squashing of lower abdomen against the floor, and 4) abdominal retractions, were each counted as 1.

#### Statistical Analysis

Statistical and curve-fitting analyses were performed using Prism 4.0 (GraphPad Software Inc., La Jolla, CA, USA). The data are expressed as means ± standard deviation. Student's t-test compared treatment means with control means. Two-sided P-values≤0.05 were statistically significant.

## Results

### Phase 1: Epidemiological Studies

**Figure 1:**
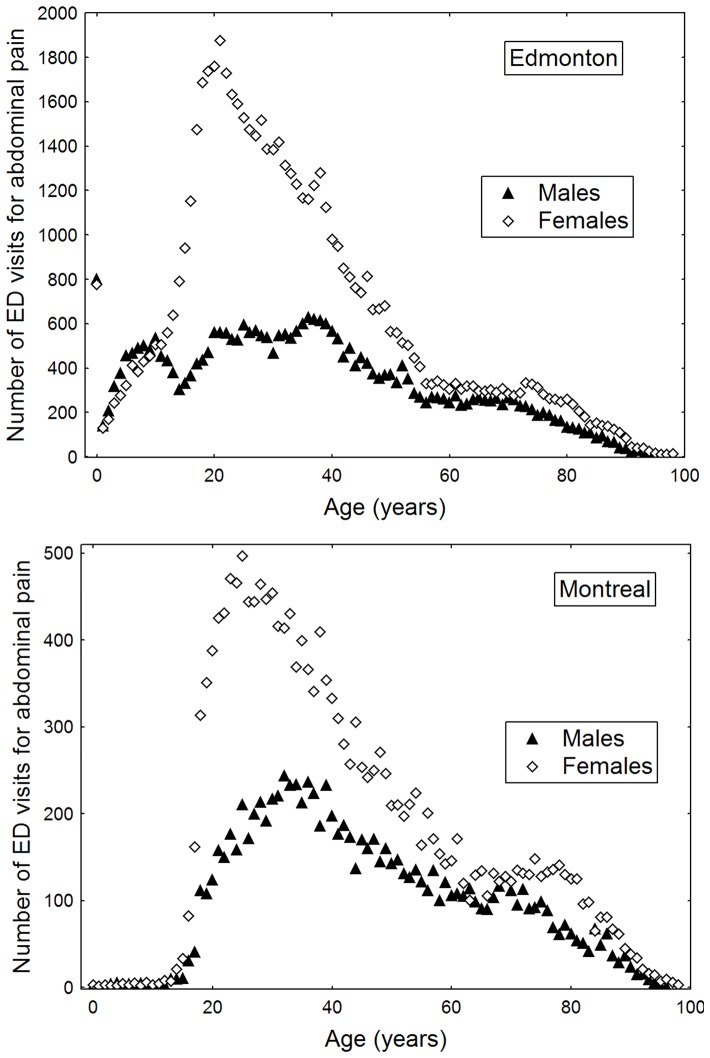
Age-specific frequency of admissions to emergency departments (ED) for non-specific abdominal pain stratified by sex.

Nearly 2/3 of emergency department visits for non-specific abdominal pain were by women and the majority of women presented in adolescence and early adulthood (Figure 1A and 1B; Table S1). In Edmonton, where pediatric emergency department visits were available, 61.4% of emergency department visits for non-specific abdominal pain occurred before the age 34 years. In Montreal, patients between the ages of 15 and 34 years accounted for 36.7% of emergency department visits (Table S1).

Table S2 describes the mean daily concentrations and variance of each air pollutant during the study periods. The effect of air pollution exposure was only observed when ages were stratified. In Edmonton, individuals aged 15 to 24 years were at elevated risk for presenting to the emergency department on the same day of higher concentrations of CO (OR = 1.04, 95% CI = 1.02–1.06), NO_2_ (OR = 1.06, 95% CI = 1.03–1.09), SO_2_ (OR = 1.02, 95% CI = 1.00–1.04), and PM_2.5_ (OR = 1.03, 95% CI = 1.00–1.05). Similar effects were observed in Montreal for CO (OR = 1.11, 95% CI = 1.05–1.17), NO_2_ (OR = 1.09, 95% CI = 1.01–1.16), SO_2_ (OR = 1.17, 95% CI = 1.10–1.25), and PM_2.5_ (OR = 1.09, 95% CI = 1.04–1.15) (Table 1). Among 15 to 24 year olds, O_3_ was negatively associated with emergency department visits for non-specific abdominal pain in Edmonton (OR = 0.95, 95% CI = 0.91–0.99), but not in Montreal (OR = 1.03, 95% CI = 0.95–1.11) (Table 1). The effect of air pollutants on individuals aged 15 to 24 stratified by sex are presented in Table 2. Interaction terms were not significant between sexes (Table 2). The effect of air pollutants with 1-day and 2-day lags stratified by age and sex are presented in Tables S3 and S4, respectively.

**Table 1:** Odds ratio for presenting to an emergency department with non-specific abdominal pain associated with an interquartile increase in an air pollutant concentration on the same day of ED visit stratified by age.

Pollutant	All Ages OR (95% CI)	<15 years OR (95% CI)	15–24 years OR (95% CI)	25–34 years OR (95% CI)	35–44 years OR (95% CI)	45–64 years OR (95% CI)	>64 years OR (95% CI)
*Edmonton*							
CO (ppm)	1.01(0.99–1.02)	0.99(0.97–1.02)	1.04(1.02–1.06)	0.99(0.97–1.01)	1.00(0.98–1.02)	1.01(0.99–1.03)	1.01(0.99–1.04)
NO_2_ (ppb)	1.02(1.00–1.03)	1.00(0.97–1.04)	1.06(1.03–1.09)	0.99(0.96–1.01)	1.01(0.98–1.04)	1.03(0.99–1.06)	1.00(0.96–1.04)
SO_2_ (ppb)	1.01(1.00–1.02)	1.00(0.98–1.03)	1.02(1.00–1.04)	0.99(0.97–1.02)	1.00(0.98–1.02)	1.00(0.97–1.02)	1.04(1.01–1.07)
O_3_ (ppb)	0.99(0.97–1.01)	0.98(0.93–1.03)	0.95(0.91–0.99)	1.02(0.98–1.06)	1.02(0.98–1.07)	0.94 (0.90–0.98)	0.99(0.94–1.04)
PM_10_ (mg/m^3^)	1.01(1.00–1.02)	1.01(0.98–1.04)	1.04(1.02–1.07)	1.01(0.98–1.03)	0.99(0.97–1.02)	1.00(0.97–1.02)	1.00(0.97–1.03)
PM_2.5_ (mg/m^3^)	1.00(0.99–1.01)	1.00(0.97–1.04)	1.03(1.00–1.05)	0.99(0.96–1.02)	0.99(0.96–1.02)	1.00(0.97–1.03)	0.99(0.96–1.03)
*Montreal*							
CO (ppm)	1.02(0.99–1.04)	NA	1.11(1.05–1.17)	1.04(1.00–1.09)	0.99(0.94–1.04)	1.08(1.04–1.13)	1.01(0.96–1.07)
NO_2_ (ppb)	1.02(0.99–1.04)	NA	1.09(1.01–1.16)	1.06(1.00–1.12)	1.00(0.94–1.06)	1.14(1.08–1.20)	1.05(0.99–1.11)
SO_2_ (ppb)	1.02(1.00–1.05)	NA	1.17(1.10–1.25)	1.08(1.03–1.14)	1.02(0.96–1.08)	1.13(1.08–1.19)	1.08(1.02–1.14)
O_3_ (ppb)	0.99(0.97–1.02)	NA	1.03(0.95–1.11)	1.04(0.98–1.11)	1.09(1.02–1.16)	0.98(0.93–1.04)	1.03(0.96–1.09)
PM_10_ (mg/m^3^)	1.01(0.98–1.04)	NA	1.03(0.95–1.11)	1.05(0.98–1.12)	0.97(0.91–1.04)	1.08(1.02–1.15)	0.98(0.91–1.05)
PM_2.5_ (mg/m^3^)	1.01(0.99–1.03)	NA	1.10(1.04–1.15)	1.06(1.01–1.10)	1.03(0.99–1.08)	1.08(1.04–1.12)	1.00(0.97–1.05)

NA – not available; CO- carbon monoxide; NO_2_ – nitrogen dioxide; SO_2_ - sulphur dioxide; O_3_ - ozone; PM_10_ - particulate matter <10 microns; PM_2.5_ - particulate matter <2.5 microns; PPM - parts per million; PPB - parts per billion; mg/m^3^ - micrograms per meters cubed.

**Table 2:** Odds ratio for presenting to an emergency department with non-specific abdominal pain associated with an interquartile increase in an air pollutant concentration on the same day of ED visit stratified by gender among individuals aged 15–24 years.

	Edmonton			Montreal		
Pollutant (unit)	Males OR (95% CI)	Females OR (95% CI)	p-Value for Interaction	Males OR (95% CI)	Females OR (95% CI)	p-Value for Interaction
CO (ppm)	0.99(0.95–1.03)	1.05(1.03–1.08)	0.05	1.09(0.96–1.24)	1.10(1.03–1.18)	0.57
NO_2_ (ppb)	1.00(0.94–1.06)	1.08(1.04–1.11)	0.07	1.06(0.91–1.22)	1.09(1.01–1.18)	0.10
SO_2_ (ppb)	1.02(0.98–1.06)	1.03(1.00–1.05)	0.78	1.16(1.03–1.31)	1.14(1.07–1.23)	0.85
O_3_ (ppb)	1.01(0.93–1.09)	0.93(0.89–0.98)	0.18	1.03(0.90–1.20)	1.02(0.93–1.12)	0.10
PM_10_ (mg/m^3^)	1.04(1.00–1.09)	1.04(1.02–1.07)	0.66	1.00(0.83–1.21)	1.02(0.93–1.11)	0.26
PM_2.5_ (mg/m^3^)	1.06(1.01–1.11)	1.02(0.99–1.05)	0.07	1.09(0.98–1.21)	1.09(1.03–1.15)	0.62

OR – odds ratio; CI – confidence interval; CO- carbon monoxide; NO_2_ – nitrogen dioxide; SO_2_ - sulphur dioxide; O_3_ - ozone; PM_10_ - particulate matter <10 microns; PM_2.5_ - particulate matter <2.5 microns; PPM - parts per million; PPB - parts per billion; mg/m^3^ - micrograms per meters cubed.

### Phase 2: *In Vitro & In Vivo* Studies

The course of body weight was not significantly different in animals treated with EHC-6802 urban particles by gavage compared to vehicle treated mice (Figure S1).

#### Gastrointestinal Motility

**Figure 2:**
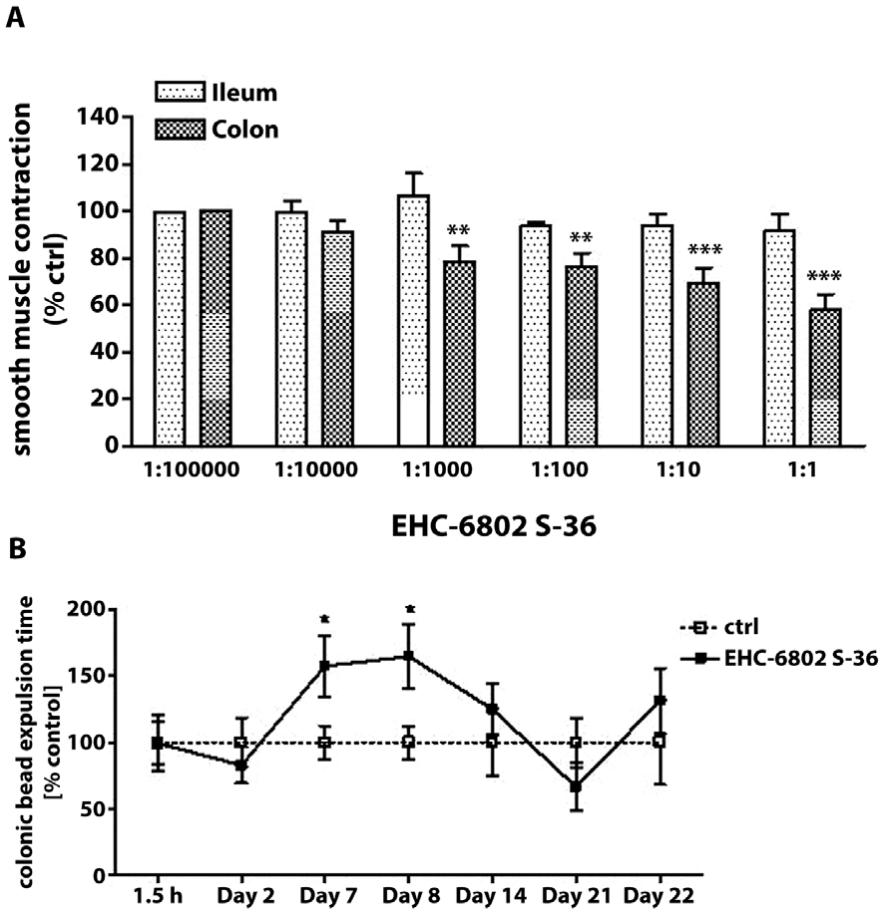
The effect of EHC-6802 on mouse GI motility. A) The effect of EHC-6802 (initial concentration 360 µg/200 µl regarded as 1∶1 dilution) on EFS (8 Hz)-stimulated smooth muscle contractions in mouse ileum and colon. Note that EHC-6802 significantly, in a concentration-dependent manner inhibited twitch contractions in mouse colon. Data represent mean ± SEM for n = 8. *P<0.05, as compared with control. B) In vivo effects of EHC-6802 (360 µg/200 µl/animal, QD, p.o.) on colonic bead expulsion time in mice. The results are shown as mean ± SEM of n = 5–10 mice for each experimental group. *P<0.05, as compared with control (animals receiving tap water).

EHC-6802 (1∶100000–1∶1) produced a significant, concentration-dependent inhibitory effect on the EFS (8 Hz)-induced twitch contractions in the colon, but not the mouse ileum (Figure 2A). Electrically induced contractions were not changed at any time points (90 min, 7 days, 21 days), compared to bethanechol (10^−6^ M) treatment (Figure S2). Responses to direct muscular activation with bethanechol (10^−7^ M–3×10^−5^ M) were not changed by adding EHC-6802 particles to the organ bath (Figure S1C) at any time point (Figure S2).

In the colonic expulsion test a 7-day treatment with EHC-6802 particles significantly reduced the time to bead expulsion (Figure 2B). Neither acute (1.5 h) nor chronic (21 days) treatment with EHC-6802 significantly affected the colonic bead expulsion time.

#### Nociception

**Figure 3:**
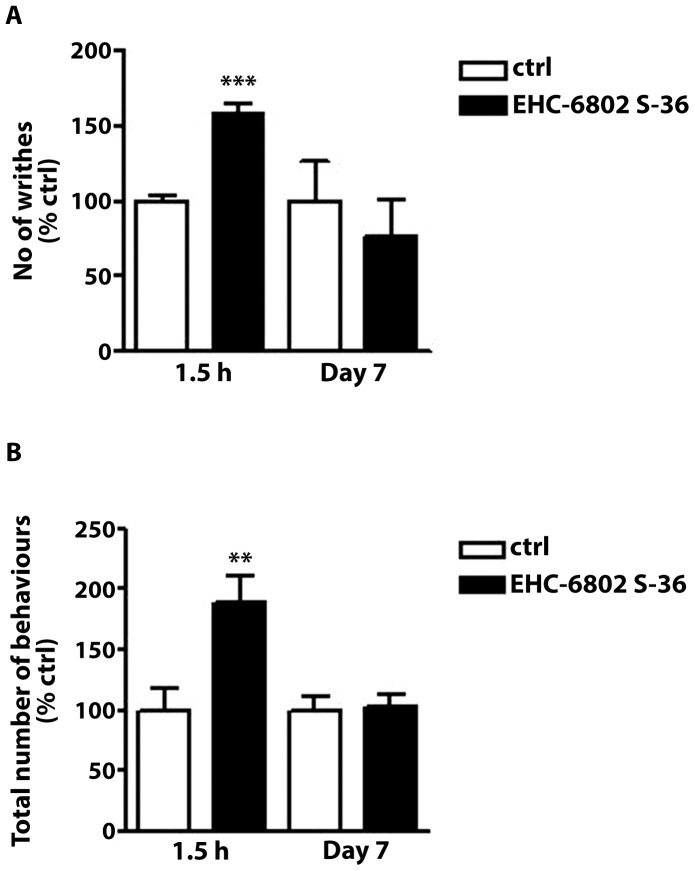
The effect of EHC-6802 (360 µg/200 µl/animal, p.o.) on behavioural pain responses in mice. A) The effect of EHC-6802 in the writhing test in mice. The number of writhes was determined 5 min after acetic acid injection (0.5%, 10 ml/kg, i.p.) over a period of 15 min. Note that a single 1.5 h- treatment with EHC-6802 significantly increased the total number of writhes. Data represent mean ± SEM of 5–10 mice per group. ***P<0.001, as compared to control (animals receiving tap water). B) The effect of EHC-6802 on the number of pain-related behaviours (licking of abdomen, stretching, squashing, abdominal contractions) evoked by i.c. administration of oil of mustard (1% in 70% EtOH - 30% saline), determined over a period of 20 min. Note that a single 1.5 h- treatment with EHC-6802 significantly increased the number of pain-related behaviours. Data represent mean ± SEM of 5–10 mice per group. **P<0.01, as compared to control (animals receiving tap water). C) The effect of EHC-6802 on the number of pain-related behaviours (licking of abdomen, stretching, squashing, abdominal contractions) evoked by the i.p. administration of neostigmine (2.5 µg/kg), determined over a period of 10 min. Note that a single 1.5 h- treatment with EHC-6802 significantly increased the number of pain-related behaviours. Data represent mean ± SEM of 5–10 mice per group. **P<0.01, as compared to control (animals receiving tap water).

In our preliminary experiments, we confirmed that the i.p. injection of acetic acid solution, i.c. instillation of OM, and the i.p. injection of neostigmine induced pain behaviors in naïve mice (data not shown). The acute administration of EHC-6802 (1.5 h treatment) significantly increased the number of writhes compared to control group (Figure 3A). 7-day (Figure 3A) and 21-day (data not shown) treatment with EHC-6802 did not modify the response to acetic acid. EHC-6802 administered at 1.5 hour also showed a significantly higher number of spontaneous pain-related behaviours induced by OM (Figure 3B) and neostigmine (Figure 3C), compared to mice after 7- and 21-day treatment (21 days, data not shown).

## Discussion

Non-specific abdominal pain is a common clinical presentation to the emergency department, typically involves multiple investigations, and may require admission to hospital. [1] A cause is often not discovered during emergency department visit and in most cases a subsequent organic diagnosis is not made during outpatient follow-up. [3] Although non-specific abdominal pain is a common disorder of the young [31], [32], triggers of acute pain are not known. We found that young individuals had the highest prevalence of non-specific abdominal pain and were significantly more likely to visit the emergency department for non-specific abdominal pain when indicators of air pollution were elevated. Furthermore, ingestion of EHC-6802 urban ambient particles by mice led to an acute exacerbation of pain, which did not persist after chronic exposure of the pollutants to the animal. These observations suggest a novel association between air pollution exposure and exacerbation of abdominal pain.

In both Edmonton and Montreal, CO, PM_2.5_, SO2, and NO_2_ were associated with non-specific abdominal pain presentations in individuals aged 15 to 24 years. However, PM_10_ was positively associated with non-specific abdominal pain in Edmonton, but null in Montreal. Furthermore, ozone was negatively associated with non-specific abdominal pain in Edmonton, but not in Montreal. Additionally, the risk estimates were generally higher in Montreal than in Edmonton. The heterogeneity of findings between cities may be explained by residents living and/or working in downtown Montreal having greater exposure to air pollutants. In contrast, residents living throughout Edmonton would have been exposed to a wider range of air pollution concentrations. The greater patient-level variability to air pollution exposure experienced in Edmonton may have resulted in non-differential misclassification that would have the effect of biasing the risk estimates towards the null. Furthermore, the fixed monitoring stations are located to represent the background air pollution concentrations in a city; however, misclassifying the air pollution exposure may occur when multiple fixed monitoring sites are averaged into one daily value for the entire city. [22] Thus, future multi-city studies are required to confirm the associations observed in our study and to explore causes of heterogeneity.

Young women are known to have the highest prevalence of non-specific abdominal pain. [31], [32] In both Edmonton and Montreal, women aged 15 to 24 years were more likely to visit an emergency department for non-specific abdominal pain. The reason for the disproportionate occurrence of non-specific abdominal pain in women as compared to men is not clear. Abdominal pain may be exacerbated during the menstrual cycle. [33] Furthermore, women have been shown to be more likely to seek medical attention for abdominal pain when compared to men. [34], [35] We did not observe effect modification between men and women aged 15 to 24 years, suggesting that the effect of air pollution on non-specific abdominal pain was not sex-specific. However, because the risk estimates observed in our study were modest, we may have not been adequately powered to detect effect modification by sex.

The mechanism by which air pollutants may exacerbate abdominal pain is not known. In one study high concentrations of air pollutants (NO_2_, CO, and O_3_) impaired gastric basal contractility. [36] However, low-level exposures were not associated with gastric dysmotility. [36] Another study demonstrated that animals fed EHC-6802 had increased IL-8 secretion from the small bowel and experienced changes in the composition of colonic microflora. [37] Our *in vitro* studies demonstrated impaired colonic, but not ileal, contractility after the acute exposure of the ex-vivo tissue with the particulate matter. Neuronally mediated contractility was impaired whereas the direct smooth muscular reactivity was unchanged suggesting that the observed effects involved neuronal rather than muscular mechanisms. *In vivo* reduction of colonic motility was not observed acutely, but a significantly slowed colonic motility was recorded after 7 days, but not after 21 days of daily exposure to the EHC-6802 particles. Overall *in vitro* and *in vivo* effects on intestinal motility by acute and chronic exposure to EHC-6802 particles were negligible.

In contrast, acute exposure to EHC-6802 particles led to significantly increased pain response in mice and this observation was consistent in 3 different behavioral tests of visceral abdominal pain. Chronic exposure to ingested pollutants did not affect the pain response models suggesting that pain adaptation to the pollutants may have occurred over time. The cause for the acute hypersensitivity in mice is not known. The animal studies suggest that the pain response following acute exposure to the pollutants were not driven by intestinal dysmotility. Exposure to air pollution may exacerbate systemic inflammation [38] or lead to oxidative damage of colonic mucosa. [39] Furthermore, air pollution has been shown to mediate dysfunction of endothelial-dependent vasodilation [40] that may contribute to visceral hypersensitivity. Future studies will be needed to explore the exact mechanisms and the associated neuronal circuits involved in triggering abdominal pain.

Several limitations should be considered. First, regional estimates of air pollutants from fixed monitoring stations may not reflect the variability of patient-level exposure to air pollutants. Though, the effects of air pollutants were likely underestimated as measurement errors were presumably non-differential. Second, we attempted to control for confounding through the use of a case crossover study design whereby cases served as their own control; however, residual confounding may have influenced our estimates. For example aeroallergens, which may correlate with air pollution [41], were not evaluated in this study; however, aeroallergens are not known to trigger non-specific abdominal pain. Third, associations were explored in multiple pollutants using several lagged exposures, and stratified to investigate several factors (e.g. age groups). Thus, the probability of observing statistically significant findings by chance is increased. We attempted to minimize multiple comparison errors by including a replication study population and primarily reporting on associations observed in both populations. However, future studies are necessary to confirm our findings. Fourth, some of the non-specific abdominal pain patients may have had an unrecognized organic disease that was diagnosed after discharge from the emergency department. For example, NO_2_ exposure (i.e., traffic pollutant) was associated with the development of appendicitis. [15] Furthermore, individuals less than 24 years and living in areas of higher concentration of NO_2_ were at a two-fold increased risk of developing Crohn's disease. [16] Because our risk estimates were modest in size, our findings may reflect significant associations among a specific gastrointestinal disorder (e.g., Crohn's disease). Nonetheless, discovering a possible link between air pollution exposure and gastrointestinal disease is an important finding that should motivate further research. Finally, while EHC-6802 particles have been validated to represent daily city exposure of air pollutants [26], our findings should be interpreted cautiously because EHC-6802 particles were gathered in Ottawa and consists of multiple air pollutants.

In conclusion, individuals aged 15 to 24 years were most likely to present to emergency departments with non-specific abdominal pain and this occurred more frequently when the concentration of CO, PM_2.5_, SO2, and NO_2_ were elevated in the atmosphere. These findings were observed in a population-based study in Edmonton and replicated in an urban downtown emergency department in Montreal. Additionally, we demonstrated that acute exposure to ingested EHC-6802 particles exacerbated abdominal pain in mice, a response that led to adaptation after chronic exposure. The consistency of the epidemiological and animal studies suggests that air pollution may trigger non-specific abdominal pain in some cases. Thus, future patient-level and basic laboratory studies should be performed to substantiate the findings, further explore possible underlying mechanisms, and identify at risk populations.

## Supporting Information

Figure S1 A) In vivo effects of EHC-6802 (360 µg/200 µl/animal, QD, p.o.) or tap water (200 µl/animal, QD, p.o.) during a 21 day treatment course. B) EFS (8 Hz)-stimulated smooth muscle contractions in mouse colon were not changed following 90 min, 7 days or 21 days of treatment with EHC-6802 S-36, as compared to vehicle treated mice. C) Bethanechol (10^−7^ M–3×10^−5^ M) stimulated smooth muscle contractions in mouse ileum (left) and colon (right) were not changed when EHC-6802 (360 µg/200 µl) was added to the organ bath.
(TIF)

Figure S2 **Bethanechol (10^−7^ M–3×10^−5^ M) stimulated smooth muscle contractions in mouse ileum and colon were not changed following 90 min, 7 days or 21 days of treatment with EHC-6802 S-36, as compared to vehicle treated mice.**
(TIF)

Table S1 **Frequency of emergency department visits for non-specific abdominal pain by age group and gender, Edmonton (EDM) and Montreal (MON).**
(DOCX)

Table S2 **Frequency distribution of the daily concentrations of ambient air pollutants and meteorological factors from April 01, 1992 to March 31, 2002 in Edmonton and January 01, 1997 to December 31, 2002 in Montreal.**
(DOCX)

Table S3 **Odds ratio for presenting to an emergency department with non-specific abdominal pain associated with an interquartile increase in an air pollutant concentration stratified by age for 1- and 2-day lags.** NA – not available; CO- carbon monoxide; NO_2_ – nitrogen dioxide; SO_2_ - sulphur dioxide; O_3_ - ozone; PM_10_ - particulate matter <10 microns; PM_2.5_ - particulate matter <2.5 microns; PPM - parts per million; PPB - parts per billion; mg/m^3^ - micrograms per meters cubed.
(DOCX)

Table S4 **Odds ratio for presenting to an emergency department with non-specific abdominal pain associated with an interquartile increase in an air pollutant concentration for 1- and 2-day lag stratified by gender among individuals aged 15–24 years.** OR – odds ratio; CI – confidence interval; CO- carbon monoxide; NO_2_ – nitrogen dioxide; SO_2_ - sulphur dioxide; O_3_ - ozone; PM_10_ - particulate matter <10 microns; PM_2.5_ - particulate matter <2.5 microns; PPM - parts per million; PPB - parts per billion; mg/m^3^ - micrograms per meters cubed.
(DOCX)
