# Supplementary figures and images for: Non-Specific Abdominal Pain and Air Pollution: A Novel Association
Source: PLoS One. 2012 Oct 31;7(10):e47669. doi: 10.1371/journal.pone.0047669 (PMC3485276; doi:10.1371/journal.pone.0047669)

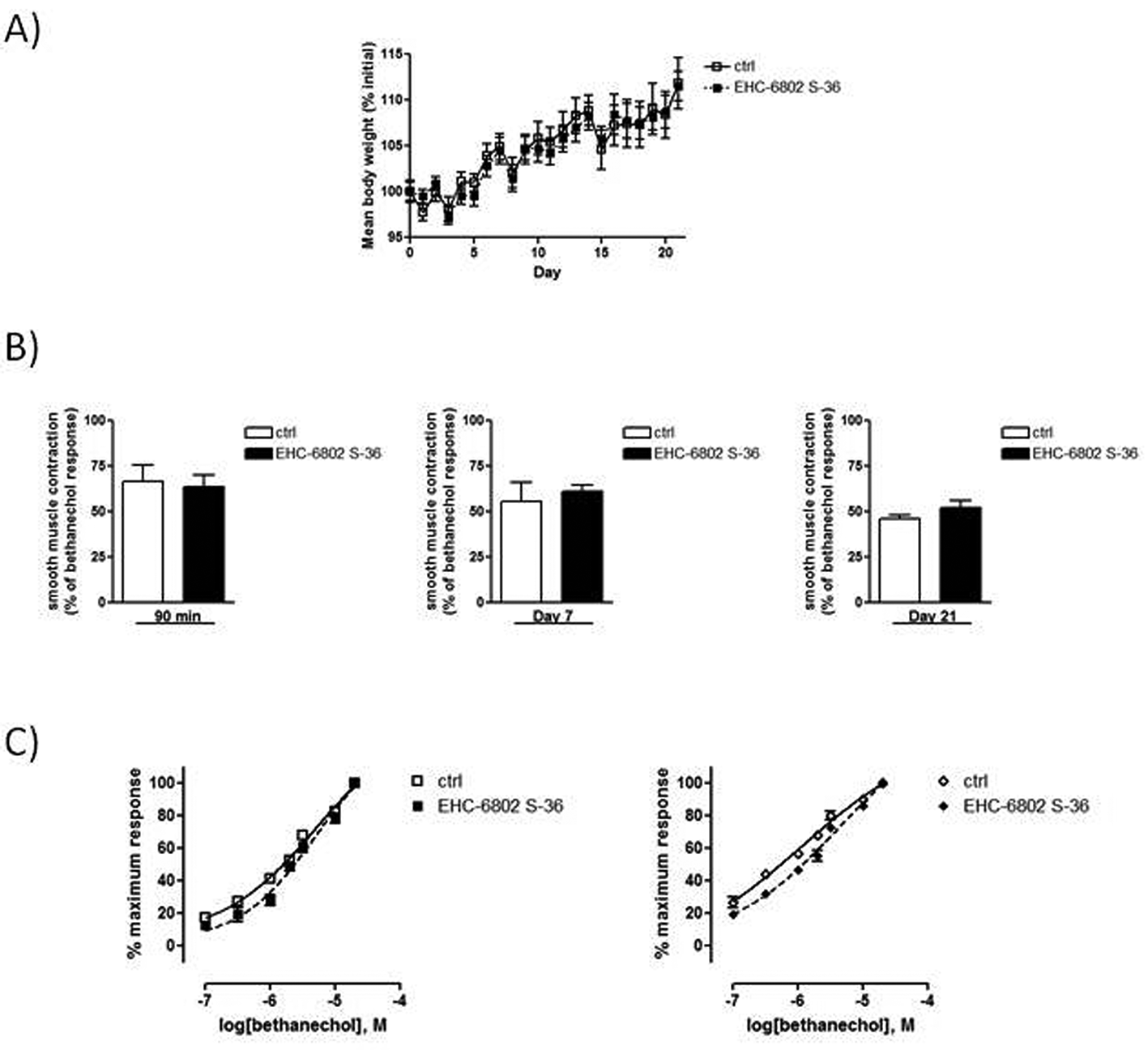

Supplement: Figure S1 — A) In vivo effects of EHC-6802 (360 µg/200 µl/animal, QD, p.o.) or tap water (200 µl/animal, QD, p.o.) during a 21 day treatment course. B) EFS (8 Hz)-stimulated smooth muscle contractions in mouse colon were not changed following 90 min, 7 days or 21 days of treatment with EHC-6802 S-36, as compared to vehicle treated mice. C) Bethanechol (10−7 M–3×10^−5^ M) stimulated smooth muscle contractions in mouse ileum (left) and colon (right) were not changed when EHC-6802 (360 µg/200 µl) was added to the organ bath. (TIF) [file pone.0047669.s001.tif]

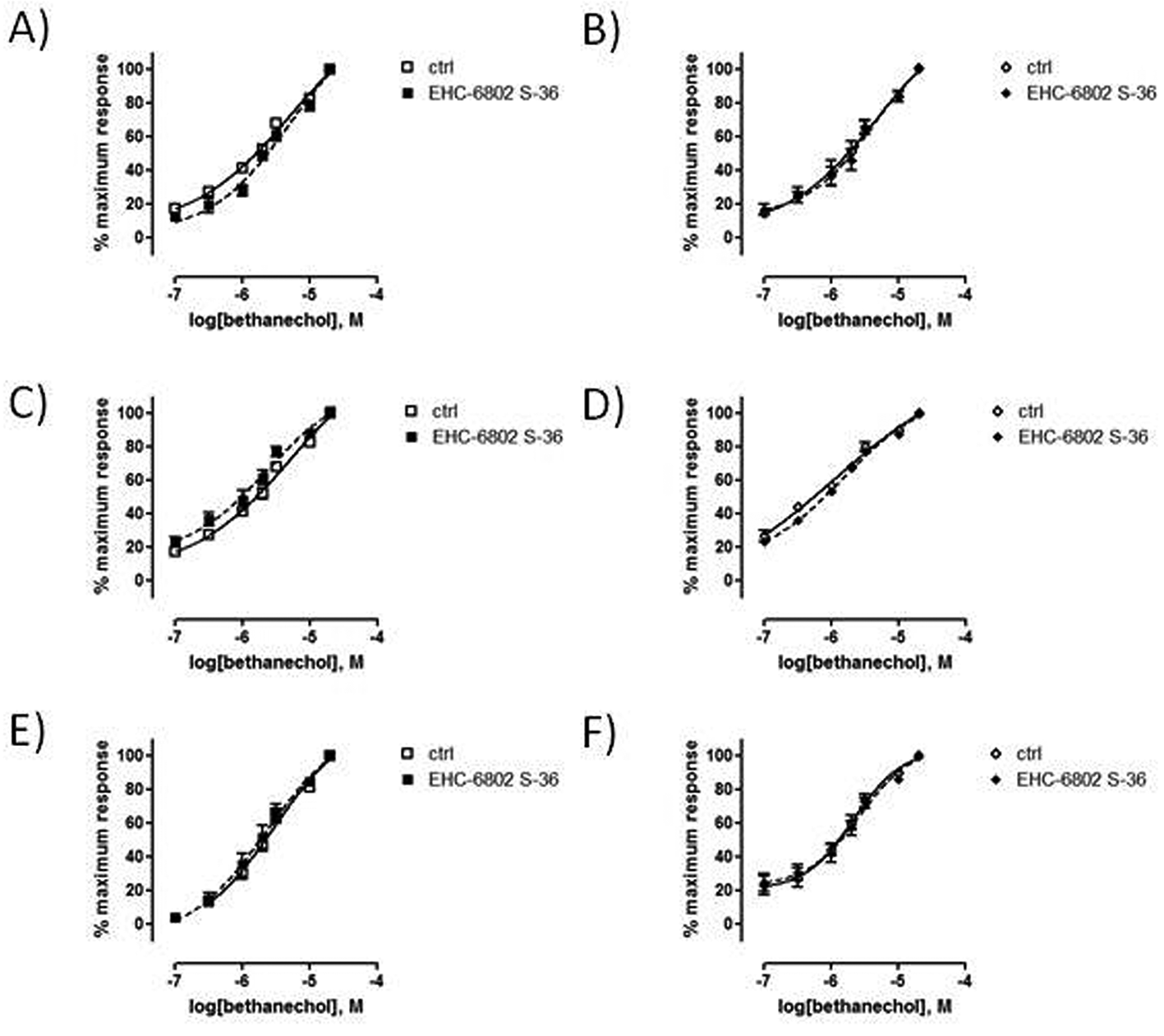

Supplement: Figure S2 — Bethanechol (10−7 M–3×10^−5^ M) stimulated smooth muscle contractions in mouse ileum and colon were not changed following 90 min, 7 days or 21 days of treatment with EHC-6802 S-36, as compared to vehicle treated mice. (TIF) [file pone.0047669.s002.tif]
