# Supplementary material for: Non-Specific Abdominal Pain and Air Pollution: A Novel Association
Source: PLoS One. 2012 Oct 31;7(10):e47669. doi: 10.1371/journal.pone.0047669 (PMC3485276; doi:10.1371/journal.pone.0047669)
Supplement: Table S1 — Frequency of emergency department visits for non-specific abdominal pain by age group and gender, Edmonton (EDM) and Montreal (MON). (DOCX) [file pone.0047669.s003.docx]

**Table S1**: Frequency of emergency department visits for non-specific abdominal pain by age group and gender, Edmonton (EDM) and Montreal (MON).

| **City:** | **Edmonton** | | | **Montreal*** | | |
| --- | --- | --- | --- | --- | --- | --- |
| **Age group** | **#EDM** | **%** | **#Female** | **#MON** | **%** | **#Female** |
| <15 | 12,954 | 19.6 | 6,594 | NA | NA | NA |
| 15 – 24 | 20,338 | 21.4 | 15,568 | 3,568 | 13.8 | 2,656 |
| 25 - 34 | 19,455 | 20.4 | 13,969 | 5,914 | 22.9 | 4,010 |
| 35 - 44 | 15,829 | 16.6 | 10,304 | 4,877 | 18.9 | 3,049 |
| 45 - 64 | 15,745 | 16.5 | 9,445 | 6,195 | 24.0 | 3,682 |
| >64 | 10,852 | 11.4 | 6,359 | 5,165 | 20.0 | 3,080 |
| **Total** | **95,173** | **100** | **62,239** | **25,852** | **100** | **16,553** |

*1,670 cases missing for Montreal (age or sex unknown) and population restricted to individuals 15 years or older. NA: not available
