# Supplementary material for: Non-Specific Abdominal Pain and Air Pollution: A Novel Association
Source: PLoS One. 2012 Oct 31;7(10):e47669. doi: 10.1371/journal.pone.0047669 (PMC3485276; doi:10.1371/journal.pone.0047669)
Supplement: Table S2 — Frequency distribution of the daily concentrations of ambient air pollutants and meteorological factors from April 01, 1992 to March 31, 2002 in Edmonton and January 01, 1997 to December 31, 2002 in Montreal. (DOCX) [file pone.0047669.s004.docx]

**Table S2**: Frequency distribution of the daily concentrations of ambient air pollutants and meteorological factors from April 01, 1992 to March 31, 2002 in Edmonton and January 01, 1997 to December 31, 2002 in Montreal.

| **City:** | **Edmonton** | | | |  | **Montreal** | | |
| --- | --- | --- | --- | --- | --- | --- | --- | --- |
| **Variable (unit)** | **Days** | **Mean** | **SD** | **IQR** | **Days** | **Mean** | **SD** | **IQR** |
| CO (ppm) | 3,652 | 0.7 | 0.4 | 0.4 | 2,191 | 0.5 | 0.2 | 0.2 |
| NO_2_ (ppb) | 3,652 | 21.9 | 9.4 | 12.8 | 2,191 | 19.4 | 7.6 | 9.5 |
| SO_2_ (ppb) | 3,616 | 2.6 | 1.8 | 2.3 | 2,191 | 4.8 | 3.0 | 3.4 |
| O_3_ (ppb) | 3,652 | 18.6 | 9.3 | 14.0 | 2,191 | 18.3 | 9.5 | 12.1 |
| PM_10_ (µg/m^3^) | 2,813 | 22.6 | 13.1 | 15.0 | 1,092 | 25.8 | 14.2 | 15.9 |
| PM_2.5_ (µg/m^3^) | 1,444 | 8.5 | 6.2 | 6.2 | 1,938 | 8.6 | 6.7 | 6.8 |
| Temperature (^o^C) | 3,652 | 3.9 | 11.9 | 17.9 | 2,191 | 7.7 | 11.4 | 19.0 |
| Humidity (%) | 3,652 | 66.0 | 13.6 | 18.5 | 2,191 | 70.7 | 12.5 | 17.8 |
