# Supplementary material for: Non-Specific Abdominal Pain and Air Pollution: A Novel Association
Source: PLoS One. 2012 Oct 31;7(10):e47669. doi: 10.1371/journal.pone.0047669 (PMC3485276; doi:10.1371/journal.pone.0047669)
Supplement: Table S3 — Odds ratio for presenting to an emergency department with non-specific abdominal pain associated with an interquartile increase in an air pollutant concentration stratified by age for 1- and 2-day lags. NA – not available; CO- carbon monoxide; NO2 – nitrogen dioxide; SO2 - sulphur dioxide; O3 - ozone; PM10 - particulate matter <10 microns; PM2.5 - particulate matter <2.5 microns; PPM - parts per million; PPB - parts per billion; mg/m3 - micrograms per meters cubed. (DOCX) [file pone.0047669.s005.docx]

**Table S3:** Odds ratio for presenting to an emergency department with non-specific abdominal pain associated with an interquartile increase in an air pollutant concentration stratified by age for 1- and 2-day lags. NA – not available; CO- carbon monoxide; NO_2_ – nitrogen dioxide; SO_2_ - sulphur dioxide; O_3_ - ozone; PM_10_ - particulate matter < 10 microns; PM_2.5_ - particulate matter < 2.5 microns; PPM - parts per million; PPB - parts per billion; mg/m^3^ - micrograms per meters cubed.

| **Pollutant** | **All Ages**  **OR (95% CI)** | **< 15 years**  **OR (95% CI)** | **15 – 24 years**  **OR (95% CI)** | **25 – 34 years**  **OR (95% CI)** | **35 – 44 years**  **OR (95% CI)** | **45 – 64 years**  **OR (95% CI)** | **>64 years**  **OR (95% CI)** |
| --- | --- | --- | --- | --- | --- | --- | --- |
| *Edmonton* | | | | | | | |
| CO (ppm)  1-Day Lag  2-Day Lag | 1.01(0.99-1.01)  1.01(0.99-1.02) | 1.00(0.98-1.02)  1.00(0.98-1.03) | 1.03(1.01-1.04)  1.02(1.00-1.04) | 0.98(0.96-1.00)  1.00(0.99-1.02) | 1.00(0.98-1.02)  1.01(0.98-1.03) | 1.01(0.98-1.03)  1.01(0.98-1.03) | 1.02(1.00-1.05)  1.01(0.98-1.04) |
| NO_2_ (ppb)  1-Day Lag  2-Day Lag | 1.02(0.99-1.01)  1.00(0.99-1.01) | 1.00(0.98-1.04)  1.01(0.98-1.05) | 1.04(1.01-1.07)  1.01(0.98-1.04) | 0.97(0.95-1.00)  1.00(0.97-1.03) | 1.01(0.98-1.05)  1.02(0.99-1.05) | 0.99(0.96-1.03)  0.99(0.96-1.02) | 1.03(0.99-1.07)  1.00(0.96-1.04) |
| SO_2_ (ppb)  1-Day Lag  2-Day Lag | 1.01(1.00-1.02)  1.00(0.99-1.01) | 1.01(0.99-1.03)  1.00(0.97-1.02) | 1.00(0.99-1.03)  0.99(0.97-1.01) | 1.02(1.00-1.04)  1.01(0.99-1.03) | 0.99(0.97-1.02)  1.01(0.99-1.03) | 1.01(0.98-1.03)  1.00(0.98-1.02) | 1.03(1.00-1.06)  1.01(0.99-1.04) |
| O_3_ (ppb)  1-Day Lag  2-Day Lag | 0.99(0.98-1.01)  1.00(0.98-1.02) | 0.97(0.93-1.02)  0.95(0.90-1.00) | 0.98(0.94-1.02)  1.01(0.98-1.05) | 1.01(0.98-1.06)  1.00(0.96-1.04) | 1.03(0.98-1.07)  1.04(1.00-1.09) | 0.98(0.93-1.02)  0.96(0.92-1.01) | 0.96(0.91-1.01)  0.98(0.93-1.03) |
| PM_10_ (mg/m^3^)  1-Day Lag  2-Day Lag | 1.00(0.99-1.01)  1.00(0.98-1.01) | 1.01(0.98-1.04)  1.02(0.90-1.05) | 1.02(1.00-1.05)  1.00(0.98-1.02) | 0.99(0.97-1.01)  0.99(0.97-1.02) | 1.00(0.98-1.03)  1.00(0.98-1.03) | 0.97(0.95 1.00)  0.98(0.95-1.00) | 1.02(0.99-1.05)  1.02(0.98-1.05) |
| PM_2.5_ (mg/m^3^)  1-Day Lag  2-Day Lag | 1.00(0.99-1.02)  1.01(0.99-1.02) | 1.02(0.99-1.05)  1.01(0.98-1.05) | 1.03(1.01-1.06)  1.02(1.00-1.05) | 0.99(0.96-1.01)  1.00(0.97-1.03) | 1.00(0.97-1.03)  1.01(0.98-1.04) | 0.97(0.95-1.00)  1.00(0.97-1.02) | 1.02(0.98-1.05)  1.00(0.97-1.04) |
| *Montreal* | | | | | | | |
| CO (ppm)  1-Day Lag  2-Day Lag | 1.00(0.97-1.02)  0.99(0.97-1.02) | NA | 1.06(1.01-1.13)  1.04(0.97-1.11) | 1.00(0.95-1.05)  1.02(0.97-1.07) | 1.01(0.96-1.07)  1.06(1.01-1.12) | 1.02(0.98-1.07)  0.99(0.95-1.04) | 0.98(0.93-1.03)  1.00(0.95-1.06) |
| NO_2_ (ppb)  1-Day Lag  2-Day Lag | 1.00(0.98-1.03)  1.00(0.98-1.03) | NA | 1.08(1.01-1.16)  1.08(1.00-1.16) | 1.03(0.97-1.09)  1.05(1.00-1.12) | 1.01(0.96-1.08)  1.09(1.03-1.16) | 1.07(1.02-1.13)  1.03(0.97-1.08) | 0.99(0.93-1.05)  1.03(0.97-1.10) |
| SO_2_ (ppb)  1-Day Lag  2-Day Lag | 1.02(0.99-1.04)  1.01(0.99-1.04) | NA | 1.16(1.08-1.23)  1.14(1.06-1.22) | 1.06(1.01-1.12)  1.04(0.98-1.10) | 1.11(1.05-1.17)  1.12(1.06-1.19) | 1.11(1.05-1.16)  1.09(1.03-1.14) | 1.02(0.97-1.08)  1.07(1.01-1.13) |
| O_3_ (ppb)  1-Day Lag  2-Day Lag | 0.99(0.96-1.02)  1.00(0.98-1.03) | NA | 1.09(1.01-1.18)  1.15(1.07-1.24) | 1.04(0.98-1.11)  1.05(0.99-1.12) | 1.05(0.98-1.12)  1.02(0.96-1.09) | 1.02(0.96-1.08)  1.09(1.03-1.16) | 1.06(0.99-1.13)  1.01(0.95-1.08) |
| PM_10_ (mg/m^3^)  1-Day Lag  2-Day Lag | 0.98(0.95-1.01) 0.99(0.96-1.02) | NA | 0.97(0.89-1.05)  1.03(0.94-1.13) | 1.00(0.93-1.07)  1.02(0.95-1.10) | 0.97(0.91-1.04)  1.00(0.93-1.08) | 1.00(0.94-1.07)  0.99(0.93-1.06) | 0.98(0.92-1.05)  0.99(0.92-1.07) |
| PM_2.5_ (mg/m^3^)  1-Day Lag  2-Day Lag | 1.00(0.98-1.02)  1.01(0.99-1.03) | NA | 1.08(1.03-1.14)  1.09(1.04-1.15) | 1.04(1.00-1.08)  1.05(1.01-1.09) | 1.05(1.00-1.09)  1.09(1.04-1.13) | 1.04(1.00-1.08)  1.03(0.99-1.07) | 0.98(0.94-1.03)  1.01(0.97-1.06) |
