# Supplementary material for: Non-Specific Abdominal Pain and Air Pollution: A Novel Association
Source: PLoS One. 2012 Oct 31;7(10):e47669. doi: 10.1371/journal.pone.0047669 (PMC3485276; doi:10.1371/journal.pone.0047669)
Supplement: Table S4 — Odds ratio for presenting to an emergency department with non-specific abdominal pain associated with an interquartile increase in an air pollutant concentration for 1- and 2-day lag stratified by gender among individuals aged 15–24 years. OR – odds ratio; CI – confidence interval; CO- carbon monoxide; NO2 – nitrogen dioxide; SO2 - sulphur dioxide; O3 - ozone; PM10 - particulate matter <10 microns; PM2.5 - particulate matter <2.5 microns; PPM - parts per million; PPB - parts per billion; mg/m3 - micrograms per meters cubed. (DOCX) [file pone.0047669.s006.docx]

**Table S4:** Odds ratio for presenting to an emergency department with non-specific abdominal pain associated with an interquartile increase in an air pollutant concentration for 1- and 2-day lag stratified by gender among individuals aged 15-24 years. OR – odds ratio; CI – confidence interval; CO- carbon monoxide; NO_2_ – nitrogen dioxide; SO_2_ - sulphur dioxide; O_3_ - ozone; PM_10_ - particulate matter < 10 microns; PM_2.5_ - particulate matter < 2.5 microns; PPM - parts per million; PPB - parts per billion; mg/m^3^ - micrograms per meters cubed.

|  | **Edmonton** | | **Montreal** | |
| --- | --- | --- | --- | --- |
|  | **Males**  **OR (95% CI)** | **Females**  **OR (95% CI)** | **Males**  **OR (95% CI)** | **Females**  **OR (95% CI)** |
| CO (ppm)  Lag 1 Day  Lag 2 Day | 0.99(0.95-1.03)  0.96(0.92-1.00) | 1.04(1.01-1.06)  1.04(1.02-1.06) | 1.02(0.90-1.14)  1.07(0.94-1.21) | 1.07(1.01-1.15)  1.04(0.97-1.12) |
| NO_2_ (ppb)  Lag 1 Day  Lag 2 Day | 1.00(0.94-1.05)  0.94(0.89-1.00) | 1.05(1.02-1.08)  1.03(1.00-1.06) | 0.99(0.86-1.14)  1.02(0.88-1.18) | 1.10(1.02-1.19)  1.10(1.01-1.20) |
| SO_2_ (ppb)  Lag 1 Day  Lag 2 Day | 0.98(0.94-1.02)  0.96(0.92-1.00) | 1.01(0.99-1.04)  1.00(0.98-1.02) | 1.29(1.15-1.46)  1.14(1.01-1.29) | 1.11(1.03-1.19)  1.15(1.06-1.24) |
| O_3_ (ppb)  Lag 1 Day  Lag 2 Day | 1.04(0.96-1.12)  1.12(1.03-1.21) | 0.97(0.92-1.01)  0.98(0.94-1.03) | 1.18(1.02-1.37)  1.10(0.95-1.27) | 1.08(0.98-1.18)  1.15(1.05-1.26) |
| PM_10_ (mg/m^3^)  Lag 1 Day  Lag 2 Day | 1.02(0.97-1.07)  0.97(0.92-1.02) | 1.02(1.00-1.05)  1.01(0.98-1.04) | 0.96(0.81-1.14)  1.06(0.90-1.26) | 0.96(0.87-1.06)  1.04(0.94-1.16) |
| PM_2.5_ (mg/m^3^)  Lag 1 Day  Lag 2 Day | 1.02(0.97-1.07)  1.01(0.96-1.07) | 1.04(1.01-1.07)  1.02(0.99-1.05) | 1.05(0.95-1.17) 1.08(0.97-1.20) | 1.09(1.03-1.16)  1.10(1.04-1.17) |
